# Multi-drug resistant surgical site infections following caesarean delivery: a prospective observational study from urban Uganda

**DOI:** 10.1186/s12905-025-04101-7

**Published:** 2025-10-30

**Authors:** Leah Mbabazi, Sarah Hahn, Lawrence Sserubiri, Patience Natukunda, Amusa Wamawobe, Henry Kajumbula, Vivian Nakate, Christoph Lübbert, Joseph Musaazi, Hawah Nabajja, Holger Stepan, Sara Nsibirwa, Amrei von Braun

**Affiliations:** 1 Infectious Diseases Institute, College of Health Sciences, Makerere University, Kampala, Uganda; 2 Division of Infectious Diseases and Tropical Medicine, Department of Medicine I, Leipzig University Medical Center, Liebigstr. 20, Leipzig, 04103 Germany; 3 Kawempe National Referral Hospital, Kampala, Uganda; 4 Department of Medical Microbiology, Makerere University, Kampala, Uganda; 5 Department of Obstetrics, Leipzig University Medical Center, Leipzig, Germany

**Keywords:** Surgical-site infections, Caesarean section, Antimicrobial resistance, Carbapenem-resistant *Acinetobacter*, Uganda

## Abstract

**Background:**

Surgical-site infections (SSI) are a feared complication of caesarean section (CS). While the incidence of post-CS SSI is estimated at 5–10% worldwide, increasing evidence suggests that women in low- and middle-income countries (LMIC) may be disproportionally affected. Due to the lack of access to routine microbiology and surveillance, data on bacterial pathogens and resistance are scarce for post-CS SSI from LMIC, and antibiotic treatment is largely empirical. Further research on common pathogens and resistance patterns is urgently needed, considering the rise of antimicrobial resistance (AMR) especially in sub-Saharan Africa.

**Methods:**

This prospective observational study aimed to describe the bacterial pathogens, resistance profiles and clinical outcomes of women with post-CS SSI at the largest national maternity hospital in Kampala, Uganda. Women with clinical signs of post-CS SSI were eligible for inclusion. Blood and deep wound swab cultures were taken for bacterial species identification and antibiotic susceptibility testing upon enrolment. Clinical follow-up assessments were conducted on days 2, 4 and at discharge.

**Results:**

From May 2021 to January 2022, 70 women were screened for study eligibility, of which 66 were enrolled and completed follow-up. The median age was 25 years (IQR 21–29), and 5 (7.6%) participants were HIV-infected. The median time between CS and diagnosis of SSI was 12.2 days (IQR 8–15). The majority of women required surgical treatment, predominantly debridement (*n* = 51, 82%). A total of 80 bacterial pathogens were identified from deep wound swabs, of which the majority were Gram-negative (*n* = 66, 82.5%). The most frequently isolated pathogens were *Acinetobacter* spp. (*n* = 26, 32.5%), *Escherichia* (*E*.) *coli* (*n* = 18, 22.5%), and *Klebsiella* spp. (*n* = 15, 18.8%), the majority of which were highly resistant to third-generation cephalosporins (94–100%). Additionally, a large proportion of *Acinetobacter* spp. isolates were resistant to carbapenems (65.4%), gentamycin (90.9%), and quinolones (85.0%). Blood cultures were negative for bacterial growth in all participants. After a median 10.7 days (IQR 5–14) of in-patient treatment, the majority of study participants made a complete recovery (*n* = 59, 89.4%).

**Conclusions:**

Deep wound swabs from post-CS SSI predominantly identified multidrug resistant Gram-negative pathogens with limited antibiotic treatment options in this setting. Especially the large proportion of carbapenem-resistant *Acinetobacter* spp. may indicate hospital-acquisition of the infections and warrants the strengthening of infection prevention and control measures, as well as antibiotic stewardship.

## Background

Caesarean sections (CS) are among the most frequently performed surgical procedures worldwide, accounting for more than 20% of all childbirths [1]. While the proportion of deliveries by CS continues to increase in high- and middle-income countries, so far approximately 5% of all childbirths in sub-Saharan Africa are caesarean deliveries according to the World Health Organization (WHO) [1]. 

A significant concern is the development of surgical site infections (SSI) following CS, which are a leading cause of morbidity and mortality among women undergoing this common procedure [2, 3]. Both incisional infection and endomyometritis following CS are associated with increased mortality, rehospitalisation, increased length of stay, and additional healthcare costs [4, 5]. 

According to the WHO, SSI account for approximately 15–20% of all hospital-acquired infections (HAIs) globally, depending on the region, healthcare setting, and types of surgeries [6]. A recent systematic review with meta-analysis on HAI in Africa found SSI to be the most common nosocomial infection in this region [7]. 

Following caesarean deliveries, SSI occur in approximately 5% to 10% worldwide [8]. While all regions are affected, reported incidence rates of post-CS SSI vary: WHO regional estimates for post-CS SSI range from 3.87% in North America to 11.91% in Africa [9]. Additionally, countries in the Eastern Mediterranean (5.11%) and South-East Asia (7.04%) regions experience higher incidence rates compared to those in the European (4.42%) or Western Pacific (4.45%) regions. Available data indicates that countries with lower income and Human Development Index (HDI) levels have a higher incidence of post-CS SSIs [9]. 

In countries of sub-Saharan Africa, the rate of SSI following CS is especially high [10, 11]. A pooled analysis from 2019 reported rates of up to 15.6% [12]. Similarly, clinical studies from Ethiopia found patients undergoing CS to be at a 5- to 20-times higher risk of bacterial infections compared to patients delivering vaginally [13]. Most recently, Wekesa et al. estimated that around 5–6% patients are affected by post-CS SSIs at the largest National Referral Hospital in Kampala, Uganda, every month [14]. 

The increased incidence of post-CS SSI in these countries and other resource-limited settings in sub-Saharan Africa can be attributed to several factors, including limited resources for perioperative and postoperative infection control, inadequate antibiotic prophylaxis, and a higher occurrence of patient-related risk factors such as uncontrolled diabetes or malnutrition [9]. Additionally, shortages of experienced surgeons and well-equipped healthcare facilities have been identified by WHO as contributing factors [6]. 

At the same time, sub-Saharan Africa is disproportionately affected by the global challenges posed by increasing antimicrobial resistance (AMR) [15]. The development and spread of AMR is largely driven by the inappropriate use of antimicrobials, as well as insufficient infection prevention and control measures especially in healthcare settings [16]. In 2019, almost 5 million deaths were directly attributed to AMR, with the highest burden reported from western sub-Saharan Africa with 27.3 deaths per 100,000 [17]. 

Skin and soft-tissue infections range among the top six infectious syndromes dominating the global burden associated with AMR [17, 18]. Especially in HAI, such as post-CS SSI, the possibility of resistance to empirical treatment needs to be taken into account and actively screened for if clinically suspected. While the most common causative pathogen found in post-CS SSI globally is *Staphylococcus aureus* as part of the natural skin flora, studies from regions across sub-Saharan Africa report a high proportion of Gram-negative pathogens in wound infections, including those with multi-drug resistance [9, 19, 20]. Treatment failure due to undetected or delayed detection of AMR in post-CS SSI impacts treatment outcomes substantially, with patients often times requiring longer treatment durations and repeated surgical interventions [19]. 

When medically indicated, CS can undoubtedly be essential, lifesaving procedures. However, understanding risk factors as well as best possible treatment options for post-operative complications such as SSI is key to reducing morbidity and mortality among affected women. We report here the clinical, surgical and microbial characteristics of women with SSI following CS from Kawempe Hospital, the largest maternity hospital in Uganda. In this setting, the majority of CS are emergency procedures, which are known to bear a higher risk of infectious complications compared to elective CS [18]. Being a low-resource setting, routine microbial diagnostics are not available, which is a challenge for two reasons: (1) accurate surveillance data on SSI from the hospital and the region remain scarce, and therefore empirical treatment recommendations are based on microbial data from high-income settings as opposed to regional data. The lack of surveillance hampers the hospital’s ability to monitor emerging resistance patterns and implement appropriate interventions. (2) treatment at Kawempe NRH remains largely empirical, as clinicians must rely on clinical judgement only rather than being able to administer targeted treatment following pathogen identification. Potential consequences include delayed adequate antibiotic treatment with the risk of uncontrolled infection, as well as the further development and spread of resistance by unnecessary broad antibiotic treatments.

Therefore, in an effort to contribute to the improvement of care, the primary objective of our study was to characterize underlying bacterial pathogens and antibiotic resistance patterns in post-CS SSI. Secondary objectives included the assessments of pre- and post-operative antibiotic treatments as well as treatment outcomes. The assessment of antibiotic treatments was of relevance for the evaluation of the rationale behind empirical treatments (as recommended by in-house guidelines), as well as the necessity to switch to a targeted treatment following microbiological results.

## Methods

This study was a nested sub-study within a prospective observational study on AMR in infected wounds acquired by trauma or burn injury, which recruited patients from Kiruddu Referral Hospital burn unit and Mulago National Referral Hospital in Kampala, Uganda. Findings from the parent study have been published elsewhere [21, 22]. Upon the acquisition of additional funding, the original study protocol was amended to include this sub-study on post-CS SSI. The study protocol amendment included the addition of a study site and the expansion of the inclusion criteria to post-operative patients. No formal sample size calculation was done. The total number of recruitments was determined by available funding for this nested sub-study.

### Study location

This study was conducted on the postnatal ward of Kawempe National Referral Hospital (NRH) in Kampala, Uganda. Kawempe NRH is the largest tertiary hospital for obstetric care in the country with approximately 25,000 births per year, of which 35–40% are delivered by CS (25–30/day) [23]. According to in-house recommendations, patients are given ceftriaxone or ampicillin IV one hour before surgery, as well as a post-operative course of antibiotics for 2–3 days following CS, most commonly with metronidazole and ceftriaxone.

Treating clinicians and nurses on the ward were integral part of the study team. All microbiology results were reviewed by the treating clinicians for patient care.

### Study population

Women of all age groups admitted to Kawempe NRH postnatal ward with fever and/or clinical signs of SSI after caesarean delivery were eligible for inclusion, irrespective of the location of caesarean delivery. Women re-admitted to the ward due to post-CS SSI as well as women receiving continuous in-patient care since CS were eligible for inclusion. Clinical signs of SSI included pus discharge from the incision site, necrotic tissue, or pelvic abscess in-line with the SSI definition of the Center for Disease Control (CDC) [24]. For patients presenting with fever post-CS in the absence of clinical signs for wound infection enrolment was postponed (exclusion criteria) until after a full evaluation for alternative reasons for fever, namely malaria and active mycobacterial infection. These women were re-assessed for inclusion on a daily basis.

### Study design

Upon giving written informed consent, participants were enrolled consecutively. At baseline, information on demographics, medical history, details of CS and SSI, as well as antibiotic treatment, was obtained. Follow-up visits were conducted on day two and four during in-patient treatment. During follow-up study visits, the clinical treatment progress was assessed, including course of symptoms, wound assessment, vital signs, type of antibiotic treatment, type of surgical treatment, and planned duration of antibiotic treatment. The final study visit was done upon discharge from the unit, which included an overall clinical outcome assessment. Clinical outcome was classified as complete recovery, minor disability (e.g. scarring, mild pain, temporary restriction in mobility), or major disability (ongoing treatment required and/or long-term impairment).

### Sample collection and laboratory analysis

Prior to sampling, purulent wounds were cleansed once with sterile 0.9% saline and cotton. Deep wound swabs were taken from the site of infection - specifically, from the tissue or fluid beneath the skin surface of the open wound - at baseline using cotton-tipped swabs according to the Levine method [25, 26]. Blood samples were collected in vacutainers for general laboratory tests (complete blood count, creatinine, and aspartate aminotransferase) to further assess systemic infection and/or sepsis, whereas 5–8 ml of sterile venous blood were collected in adults BD Bactec Plus Aerobic/F Culture vials (Becton Dickinson, USA) for blood cultures (two blood culture bottles aerobic/anaerobic per participant). Subsequently, the blood culture samples and swabs (in Amies semi-solid transport media) were transported and processed at the Makerere University Clinical Microbiology Laboratory. This is a College of American Pathologists accredited laboratory since 2018 and all tests are done following accredited laboratory Standard Operating Procedures (SOPs). The laboratory team was blinded to clinical outcomes.

Samples collected during laboratory working hours were received by the laboratory within two hours of collection at room temperature and placed in the Bactec automated incubators immediately. Samples collected at night were delivered to the laboratory early the next morning and checked for physical turbidity on arrival. All specimens that experienced a delay were sub-cultured before placement into the blood culture system despite their turbidity status, as delays in the incubation of blood cultures may slightly reduce the detection of growth by the automated blood culture system. However, this was remedied by the initial subcultures of delayed specimens and terminal subcultures of all specimens that showed no growth on the fifth and final day of incubation.

The blood cultures bottles were incubated in automated BACTEC machines, BACTEC 9050, 9120 and FX40 (Becton Dickinson, USA). The selection of a specific BACTEC machine was not predetermined but depended on operational logistics. All BACTEC positive cultures were Gram stained and sub-cultured onto MacConkey, 5% Sheep Blood (BA) and 5% Chocolate Blood agar (CBA) (Biolab Hungary and Oxoid UK). MacConkey was incubated at 37 °C in ambient air incubator for 24 h, whereas BA and CBA in the 5% carbon dioxide incubator at 37 °C up to 72 h. Blood cultures were monitored every 24 h for growth, up to 5 days until confirmed negative. The swabs were inoculated onto CBA, BA and MacConkey agar, and incubated similarly as blood culture positive plates.

Bacterial identification was based on the morphology, Gram stain and standard biochemical tests for Gram-negative identification. We conducted various biochemical tests for lactose and non-lactose fermenting Gram-negative bacilli, including oxidase, triple sugar iron (TSI), sulphur indole motility, urease, and citrate production. For Gram-positive isolates, we performed catalase, mannitol fermentation, DNase production, and coagulase tests to confirm *Staphylococcus (S.) aureus*. *Streptococcus* species, which are typically catalase-negative, underwent haemolysis testing on blood agar to determine alpha (α) haemolysis, beta (β) haemolysis, or gamma (γ) haemolysis. Additionally, we confirmed their identity based on sensitivity or resistance to optochin, bacitracin, and cotrimoxazole (SXT). Other tests included the bile-esculin and salt tolerance (growth in 6.5% NaCl).

Antimicrobial susceptibility testing (AST) was performed on Mueller-Hinton agar (MHA) using the Kirby Bauer disk diffusion method following the laboratory SOPs and the Clinical Laboratory Standards Institute (CLSI) guidelines [27]. For AST, a pure and fresh colony bacterial lawn was made on an MHA plate for non-fastidious organisms and BA agar for fastidious organisms. No more than 5 drug disks were placed on the 90 mm plate. The plates were then incubated at 35 °C to 37 °C for 24 h.

For Gram-negative isolates, ampicillin (10 µg), cefuroxime parenteral (30 µg), amoxicillin/clavulanic acid (20/10µg), gentamicin (10 µg), trimethoprim/sulfamethoxazole (1.25/23.5 µg), chloramphenicol (5 µg), ciprofloxacin (5 µg), ceftriaxone (30 µg), cefotaxime (30 µg), ceftazidime (30 µg), cefepime (30 µg), piperacillin (100 µg), piperacillin/tazobactam (100/10µg), imipenem (10 µg)/meropenem (10 µg) was tested in addition to tetracycline (30ug) for *Acinetobacter* spp.

For Gram-positive isolates, penicillin G (10 units), cefoxitin (30 µg), ceftaroline (30 µg), erythromycin (15 µg), clindamycin (2 µg), vancomycin, tetracycline (30 µg), trimethoprim-sulfamethoxazole (1.25/23.5 µg), chloramphenicol (5 µg), ciprofloxacin (5 µg), gentamicin (10 µg), amikacin (30 µg), rifampicin (5 µg), linezolid (30 µg), and teicoplanin were tested.

AMR phenotypic tests were screened using a bacterial suspension equivalent to 0.5 McFarland turbidity standards for the study of methicillin-resistant *S. aureus* (MRSA) and extended spectrum beta lactamase (ESBL) enzyme production and incubated for overnight (18–24 h) at 37 °C. MRSA was screened by use of the cefoxitin disc (10 µg) on a Mueller-Hinton agar (Oxoid, UK) and an inhibition zone diameter of 21 mm indicated an MRSA strain. ESBL production was screened using the combined disc method. Here, a disk of amoxicillin/clavulanic acid (AMC) was placed in the center of ceftazidime (30 µg), cefotaxime (30 µg), and cefepime (30 µg) at a distance of 15 mm apart on a streaked Muller-Hinton Agar. An increase in the inhibition zone diameter of greater than 5 mm for a combination disc versus ceftazidime or cefotaxime or cefepime disc alone was interpreted as ESBL-producing strain. Lastly all isolates were cryopreserved (brain heart infusion with 20% glycerol) at −80 °C until further analysis could be undertaken.

To ensure quality control, the identification and AST of each bacterial isolate was performed alongside its respective control strain (*E. faecalis* ATCC^®^ 29212, E. coli ATCC^®^ 25922, *S. aureus* ATCC^®^ 12600, *S. saprophyticus* ATCC^®^ 15305, and *K. pneumoniae* ATCC^®^ 13883 among others). All antibiotics were quality controlled every week following the CLSI guideline on routine quality testing of antibiotics discs. In addition, all new antibiotic discs were quality-controlled ATCC strains for each drug, which ensured normal documenting recommended parameters before testing on the patient samples.

As the microbiological analyses were conducted as part of clinical care, AST was performed using a stepwise approach. In line with standard clinical microbiology practice in this setting, isolates were initially tested against first-line antibiotics. Additional testing for second-line or broader-spectrum agents (e.g., amikacin) was performed only if the isolate showed resistance to the first-line options or if there was a specific clinical indication to broaden the antimicrobial coverage.

### Data collection and statistical analysis

Data was collected by a trained study nurse and securely managed using interviewer-administered structured questionnaire pre-programmed on the web-based software platform REDCap (Research Electronic Data Capture) hosted at the Infectious Diseases Institute, College of Health Sciences, Makerere University [28, 29]. Statistical data analysis was done with STATA version 14 (StataCorp, Texas, USA). For descriptive statistics, frequencies, percentages, means and standard deviations were used. Resistance profiles and distribution of pathogens by specific antibiotics were summarised using frequencies and proportions.

### Ethical approval, confidentiality and consent to participate

This study was approved by the Mulago Hospital Research and Ethics Committee (MHREC 1835), Kawempe Referral Hospital Research ethics committee, and the Uganda National Council for Science and Technology (HS 815ES). Written informed consent was obtained from all participants before enrolment in the participants’ preferred languages. Participants below the age of 18 years were recruited as minors (assent forms with parental consent). All study documents were kept in locked cabinets. Access to the source documents and database was given to authorized personnel only (members of the immediate study team) and a log of authorized personnel was stored in the trial master file. All data and study procedures were conducted in accordance to Good Clinical Principals (GCP-ICH) and regulatory guidelines. The funding agency had no role in the study design, data collection, data analysis, or writing of this manuscript.

## Results

### Study population

**Fig. 1:**
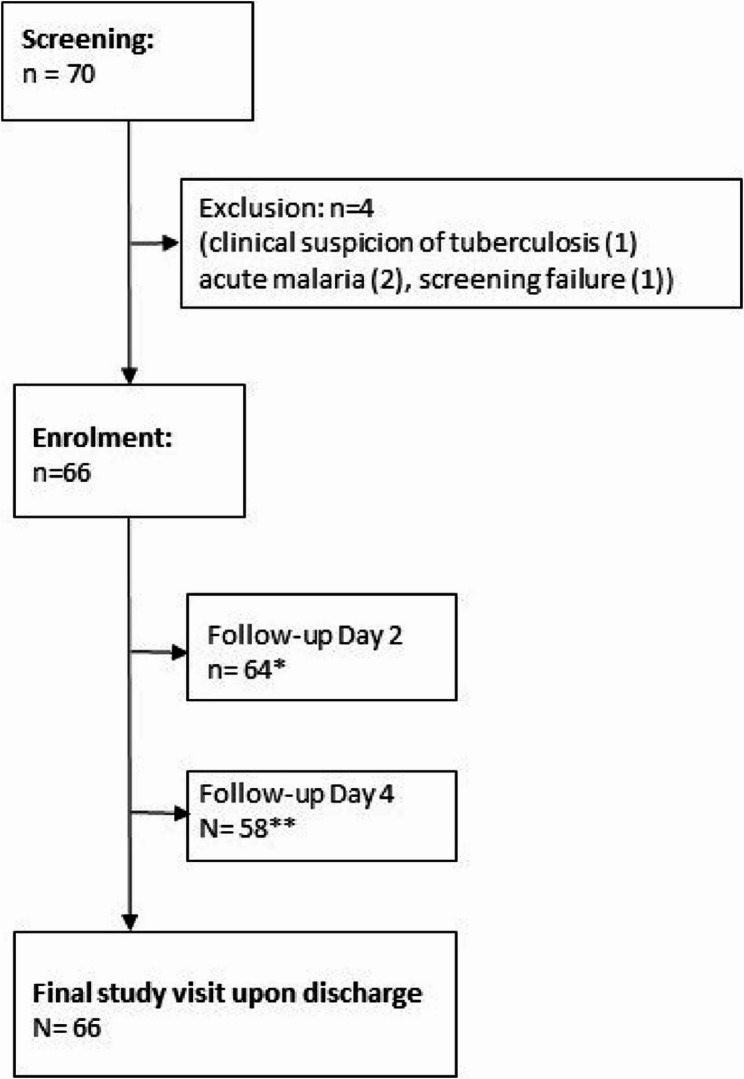
Study enrolment flowchart. *2 patients discharged before day 2, **6 patients discharged between day 2 and 4

From May 2021 to January 2022, 70 women were screened for study participation of which 66 with clinical signs of post-CS SSI were enrolled (Fig. 1).

The median age of our study population was 25 years (interquartile range (IQR) 21–29). The majority of participants had no history of chronic illness, except for one women with insulin-dependent diabetes mellitus and 5 (7.6%) participants with HIV infection, all on antiretroviral treatment. Caesarean deliveries were secondary procedures (emergency deliveries) in all cases. The majority (*n* = 53, 80.3%) presented with post-CS SSI only, a smaller proportion (*n* = 13, 19.7%) presented with concomitant fever. The median time between caesarean delivery and diagnosis of SSI was 12 days (IQR 8–15). Almost all women required surgical treatment for the SSI, predominantly debridement (*n* = 51/62, 82.3%). Furthermore, the majority (*n* = 60/66, 90.9%) were on antibiotic treatment at enrolment, most commonly with metronidazole (100%) and ceftriaxone (53.3%) in-line with in-house recommendations. Study participants´ demographics, clinical characteristics and treatment at baseline are shown in Table 1.

**Table 1:** Demographics, clinical characteristics and treatment at enrolment (*n* = 66)

Characteristics	*N* (IQR or %)
Median age in years (IQR)	25 (21–29)
HIV seropositive (%)	5 (7.6)
Median time since CS^1^ in days (IQR)	12 (8–15)
	N (%)
Antibiotic treatment in past 4 weeks	17 (25.8)
Type of antibiotic treatment in past 4 weeks^2^	
Metronidazole	17 (100.0)
Ceftriaxone	13 (76.5)
Amoxicillin	3 (17.6)
Amoxicillin and clavulanic acid	1 (5.9)
Source of antibiotic treatment in past 4 weeks	
Hospital	15 (88.2)
Drug shop/pharmacy	2 (11.8)
Health center	2 (11.8)
Abnormal vital signs	23 (34.8)
Tachycardia (> 100 bpm)	14 (60.9)
Fever (> 38 °C)	6 (26.1)
Hypertension (≥ 140/90 mmHg)	2 (8.7)
Surgical treatment	62 (93.9)
Type of surgical treatment^2^	
Debridement	51 (82.3)
Wound dressing	23 (37.1)
Exploratory laparotomy	8 (12.9)
Incision and drainage	1 (1.6)
Antibiotic treatment at enrolment	60 (90.9)
Type of current antibiotic treatment^2^	
Metronidazole	60 (100.0)
Ceftriaxone	32 (53.3)
Piperacillin/Tazobactam	19 (31.7)
Meropenem	5 (8.3)
Other antibiotics^3^	6 (10.0)

^1^missing data for 2 study participants, ^2^more than one type of surgical treatment possible, ^3^Other antibiotics: amoxicillin (*n* = 1), ampicillin + cloxacillin (*n* = 1), cefotaxime (*n* = 1), cloxacillin (*n* = 1), levofloxacin (*n* = 1), vancomycin (*n* = 1)

### Microbiological results

**Fig. 2:**
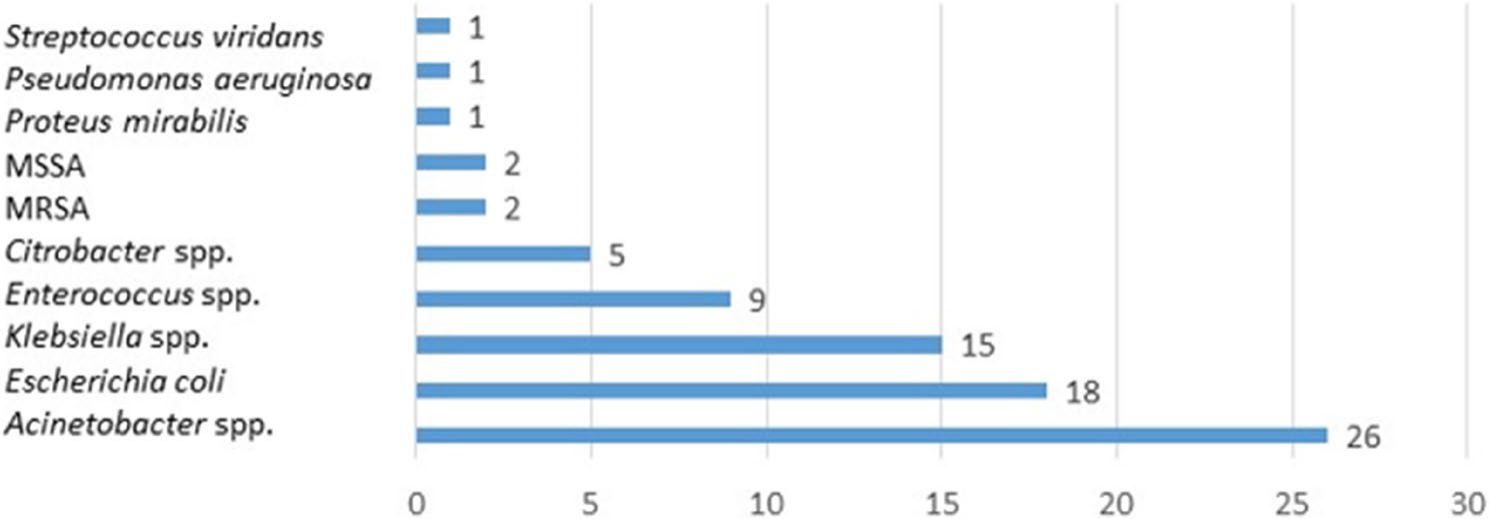
Type and number of isolates identified from wound swabs (*n* = 80). *MSSA* Methicillin-susceptible *Staphylococcus aureus, MRSA* Methicillin-resistant *Staphylococcus aureus*

Deep wound swabs revealed bacterial growth in 50 (75.8%) specimens, of which 24 (48%) showed mixed bacterial growth and 26 (52%) identified single pathogens. A total of 80 bacterial pathogens were identified, of which the majority were Gram-negative (n=66, 82.5%). The most frequently isolated Gram-negative pathogens were *Acinetobacter *spp. with 26 isolates (32.5%), followed by *Escherichia (E.) coli* with 18 isolates (22.5%), and *Klebsiella *spp*.* with 15 isolates (18.8%). Concerning the specimens with mixed bacterial growth, the majority identified two pathogens (n=19/24, 79%). The most common combinations were either *Klebsiella* spp. (n=7/19, 36%) or *E.coli* (n=5/19, 26.3%) in combination with *Acinetobacter* spp.. Among Gram-positive pathogens, *Enterococcus *spp. were identified in 9 isolates (11.3%), while *Staphylococcus (S.) aureus* including methicillin-resistant *S. aureus* (MRSA) was rarely identified (5%) (Fig. 2). Coagulase-negative Staphylococci were not identified in any wound swab. Blood cultures were negative for bacterial growth in all participants. 

Table 2 shows the proportion of most commonly identified pathogens from deep wound swabs resistant (MIC above CLSI breakpoints) to commonly used antibiotics. These three Gram-negative pathogens were almost all resistant to first choice antibiotic treatment with third generation cephalosporins (94–100%). Additionally, a large proportion of *Acinetobacter* spp. isolates were resistant to carbapenems (65.4%), gentamicin (90.9%) and quinolones (85.0%), while resistance to amikacin and colistin was rarely observed.

**Table 2:** Resistance profiles of the three most commonly identified isolates from wound swabs in patients with SSI following CS

Type of pathogen Number of isolates	Acinetobacter spp. 26 *n*/*N* (%)	E. coli 18 *n*/*N* (%)	Klebsiella spp. 15 *n*/*N* (%)
Antibiotics			
Amikacin	3/22 (13.6)	1/7 (14.3)	0/6 (0.0)
Cefotaxime/Ceftriaxone	7/7 (100.0)	16/17 (94.1)	14/14 (100.0)
Ceftazidime	22/24 (91.7)	9/12 (75.0)	13/13 (100.0)
Chloramphenicol	-	6/14 (42.9)	6/14 (42.9)
Ciprofloxacin	17/20 (85.0)	10/14 (71.4)	4/11 (36.4)
Cotrimoxazole	14/14 (100.0)	12/12 (100.0)	10/10 (100.0)
Colistin	1/9 (11.1%)	-	-
Gentamicin	20/22 (90.9)	6/16 (37.5)	5/11 (45.5)
Imipenem/Meropenem	17/26 (65.4)	1/18 (5.6)	3/15 (20.0)
Piperacillin/Tazobactam	17/21 (80.9)	1/11 (9.1)	4/7 (57.1)
Tetracycline	13/21 (61.9)	-	-

Hyphens (-) indicate not tested or intrinsically resistant, n = number of isolates resistant to a specific antibiotic, N = total number of isolates with susceptibility results for the specific antibiotic

### Treatment and outcome

The median length of in-patient treatment for SSI was 10 days (IQR 5–14). Day 2 and day 4 post-enrolment follow-up visits were completed for 64 (97%) and 58 (87.8%) participants respectively, while all 66 (100%) participants completed the final study visit upon discharge from the hospital. As assessed during the follow-up study visit on day 4 following enrolment, surgical treatment interventions included repeated debridement (*n* = 35/58, 81.4%), wound dressing (*n* = 14/58, 32.6%), and exploratory laparotomy for presumed pelvic abscess (*n* = 1/58, 2.3%). All study participants received secondary closure of the CS wound before discharge from the ward.

All study participants were treated with antibiotics throughout their hospital stay. Empirical treatment usually consisted of a combination including metronidazole (*n* = 59, 92.2%), and/or ceftriaxone (*n* = 27, 42.2%). Further common choices included piperacillin/tazobactam (*n* = 21, 32.8%) and ampicillin plus cloxacillin (*n* = 19, 29.2%). Following AST, the majority of study participants received a switch to targeted antibiotic treatment from enrolment to discharge (*n* = 56, 86.2%). The remaining participants continued initial treatment. Upon discharge, oral antibiotics were continued for a mean duration of five days in all patients.

The majority of study participants made a complete recovery (*n* = 59, 89.4%), while five (7.6%) were classified to have minor disability, and one (1.5%) major disability. One study participant died of respiratory failure on the ward due to acute SARS-CoV-2 infection.

## Discussion

This prospective study included 66 women with post-CS SSI at the largest maternity hospital in Uganda. With 12 days (IQR 8–15), the median time between CS and diagnosis of SSI was comparable to other studies on post-CS SSI from East Africa [20]. The majority of study participants were on empirical treatment with metronidazole and ceftriaxone at enrolment, which reflects internal hospital recommendations for post-CS SSI. In addition to antibiotic treatment, almost all women required surgical treatment for SSI indicating deep incisional SSI in this in-patient population rather than superficial infection [30].

As routine microbial diagnostics are unavailable in many resource-limited settings such as Uganda, data on post-CS SSI attributable pathogens and resistance rates are scarce. While the laboratory capacity, as well as the capacity to collect and report surveillance data are in place in Kampala, factors such as high costs, unreliable supply chains, and long turnaround times hamper the implementation in routine care. Strategies worth strengthening in order to improve the diagnosis and management of infections including post-CS SSI in low-resource settings include regional laboratory networks and external partnerships. Especially regional laboratory networks can overcome limitations such as lack of infrastructure, and reduce the burden on individual healthcare centers. As most recently identified by the WHO, research priorities for AMR in human health include investigations and evaluations of microbial diagnostics including conventional microbiology testing and point-of-care tests feasible for use in low-resource settings [31].

Comparable to other SSI, pathogens in post-CS SSI most commonly originate from the patient´s endogenous flora or are acquired during in-patient treatment [32]. The challenges posed by mixed bacterial growth, as shown in a large proportion of our samples, can be expected in all regions of the world, however some regional differences seem to apply. For instance, in contrast to findings from high-income settings in which Gram-positive pathogens are often found to cause post-CS SSI (e.g. *S. aureus*, group A and B *streptococci*), deep wound swabs in our study population revealed Gram-negative pathogens in the majority of cases [33]. The predominance of Gram-negative bacteria in our study population is in-line with findings from similar studies from the region. For instance, a recent prospective study conducted in rural Rwanda found Gram-negative isolates in 68.4% of post-CS SSI [20]. Available data from Uganda showed Gram-negative pathogens in 59% of post-CS SSI in April 2018; however, this has not been studied prospectively since [14]. Reasons for a predominance of Gram-negative pathogens causing post-CS SSI include selection due to available perioperative antibiotic prophylaxis with ampicillin, as well as hospital-acquisition of classical nosocomial pathogens, such as *Acinetobacter* spp [34].

In our study population, the most commonly isolated pathogen was *Acinetobacter* spp., among which especially *A. baumanii* is associated with severe nosocomial infections and hospital outbreaks across the globe [35]. In contrast to previously available data on post-CS SSI from the region, we not only found *Acinetobacter* spp. to be the most commonly isolated pathogens, the invasive isolates were also resistant to carbapenems in over 65% of all cases [14]. Of note, the WHO identified carbapenem-resistant *Acinetobacter* spp. as a “critical” no. 1 priority pathogen on its list of pathogens for which new antibiotics are urgently needed [36]. Currently, many regions in the world are experiencing rapid emergence of multidrug resistance in *Acinetobacter* spp [37]. Due to the lack of microbiological surveillance data from countries in sub-Saharan Africa, the magnitude of the problem in the region can so far only be estimated. However, increasing evidence suggests that the WHO African region is disproportionately affected by AMR including multidrug resistant *Acinetobacter* spp [17]. In-line with reports from the region, our findings suggest that urban referral hospitals may be at the centre of this development due to the increasing availability of carbapenems [38, 39]. However, by now colonization with carbapenem-resistant Enterobacterales as the most important risk factor for invasive infection with these pathogens has reached rural settings in the region as well [40, 41].

Invasive infections with carbapenem-resistant *Acinetobacter* spp. are difficult to treat even in high-income countries; however, in resource-constrained settings treatment options for this pathogen are profoundly reduced. So far, novel antibiotic combinations as available in high-resource settings are not available. It is therefore of utmost importance to curb the further development and spread of multidrug resistant *Acinetobacter* spp. by IPC measures, as well as Antimicrobial Stewardship (AMS) efforts.

Effective AMS and IPC strategies require practical, resource-sensitive measures. Targeted microbial sampling in high-risk areas like operating rooms helps identify contamination hotspots without extensive testing. Regular audits of cleaning and hygiene practices can be conducted with minimal cost. Simple, low-cost staff training on environmental surveillance and hygiene is key to improving IPC effectiveness. A multisite study conducted in East Africa demonstrated that a multimodal SSI prevention intervention can be successfully implemented when fully integrated into routine hospital practices [42].

To the best of our knowledge, this is the first prospective clinical study on post-CS SSI from Uganda in over 5 years, providing a timely and much-needed update on the current state of post-CS SSI in the region. Notably, our findings highlight the predominance of multi-drug resistant Gram-negative infections, which has become a significant concern for patient outcomes in Uganda. This pattern of resistance is consistent with global trends but is particularly relevant given the local context and the limited data available from the region. One of the novel contributions of our study is the identification of *Acinetobacter* spp. as a key pathogen involved in post-CS SSIs, which has not been widely reported in similar studies in the region. This emerging finding adds to the growing body of evidence on the role of *Acinetobacter* spp. in HAI and underscores the need for targeted surveillance and infection control measures.

In our study population, almost all invasive isolates proved to be resistant to ceftriaxone and none required anaerobic coverage provided by empirical treatment with metronidazole. However, selection of multidrug-resistant pathogens due to broad-spectrum antibiotics given in the past four weeks prior to study enrolment most likely contributed to our microbiological findings. Besides factors such as consistent availability, low costs and toxicity, the rational for adding metronidazole to the empirical treatment regimen in this setting is to cover anaerobic pathogens potentially causing concomitant postpartum endomyometritis, which at times cannot be ruled out before treatment is started. Timely clinical re-evaluation in the context of AMS would be of benefit.

To what extent our findings are generalizable to similar settings remains to be seen; however, our results do offer an idea of the dimension of multidrug-resistant Gram-negatives in this patient population and highlight the importance of targeted treatment following pathogen identification in post-CS SSI. Additionally, hospital environmental surveillance to assess the degree of contamination as a source of infection would support the development of effective countermeasures.

This study has some limitations. The sample size was determined based on the available funding, rather than a formal sample size calculation. While this approach may limit statistical power, the study provides valuable insights into the characteristics and outcomes of post-CS SSIs. The findings should be interpreted with caution, acknowledging that the lack of a formally calculated sample size may affect the generalizability of the results. Future studies with larger, power-based sample sizes are recommended to confirm and expand upon these findings. Furthermore, perioperative antibiotic prophylaxis was not assessed in detail. We can only assume that either ampicillin or ceftriaxone were used in accordance with in-house recommendations. The analysis of type and duration of perioperative antibiotic prophylaxis would have provided additional information on likely pathogen selection. If for instance ceftriaxone was commonly used with more than one or two doses administered, this may have contributed to the selection of Extended-Spectrum Cephalosporin-Resistant enterobacterales. Information on antibiotic treatment within the past 4 weeks prior to diagnosis of post-CS SSI was obtained by patient interview and is therefore subject to recall bias. Due to lack of documentation in patient files, this information could not be verified. Additionally, while it was not our intention to re-study well-known risk factors for post-CS SSI, it would have been of benefit to readers to provide more information on patient factors such as body mass index and parity, as well as on the performed CS such as midline vs. low transverse incision and other surgical factors. Furthermore, we did not assess the hospital, in which the CS was performed, nor the time spent outside of the hospital after delivery. Therefore, the origin of the SSI cannot be determined. Concerning our microbiology findings, the low number of isolates with corresponding susceptibility results limits the ability to draw conclusions concerning empirical treatment recommendations. For this, larger prospective studies are necessary. In the meantime, our results contribute to improving the knowledge gab on AMR in post-CS SSI.

## Conclusions

In our study population, almost all participants made a complete recovery following surgical and antibiotic treatment for post-CS SSI. The majority of post-CS SSI in our population were caused by multi-drug resistant Gram-negatives, especially *Acinetobacter* spp. and all women required surgical treatment due to the extent of the infection. In order to counteract the further development and spread of AMR including carbapenem-resistant *Acinetobacter* spp. the strengthening of effective IPC measures and specialized AMS teams is warranted. In recognition of the ongoing challenges to implement routine microbiological testing in this setting, we conclude that at least in patients with insufficient clinical response to empirical treatment pathogen identification and AST should be attempted and form the basis for the choice of antimicrobial treatment.

## Data Availability

The datasets used and/or analysed during the current study are available from the corresponding author on reasonable request.
